# The green microalga *Tetraselmis suecica* reduces oxidative stress and induces repairing mechanisms in human cells

**DOI:** 10.1038/srep41215

**Published:** 2017-01-24

**Authors:** Clementina Sansone, Christian Galasso, Ida Orefice, Genoveffa Nuzzo, Elvira Luongo, Adele Cutignano, Giovanna Romano, Christophe Brunet, Angelo Fontana, Francesco Esposito, Adrianna Ianora

**Affiliations:** 1 Integrative Marine Ecology Department, Stazione Zoologica Anton Dohrn, Villa Comunale, Naples 80121, Italy; 2 University of Naples “Federico II”, Department of Veterinary Medicine and Animal Production, Via Federico Delpino 1, Naples 80137, Italy; 3 Bio-Organic Chemistry Unit, Institute of Biomolecular Chemistry-CNR, Via Campi Flegrei 34, Pozzuoli, Naples 80078, Italy

## Abstract

Green microalgae contain many active pigments such as carotenoids having antioxidant and protective activity on human cells. Here we investigate the biological activity of an ethanol/water extract of the marine green microalga *Tetraselmis suecica* containing high levels of carotenoids such as the xanthophylls lutein, violaxanthin, neoxanthin, antheraxanthin and loroxanthin esters. This extract has a strong antioxidant and repairing activity in the human lung cancer cell line (A549) as shown by the increased expression of dehydrocholesterol reductase-24 (DHCR24) and prostaglandin reductase 1 (PTGR1) genes and proteins. The extract also reduces prostaglandin E_2_ (PGE_2_) levels in cells damaged by H_2_O_2_ and has tissue repairing effects on reconstructed human epidermal tissue cells (EpiDerm^TM^) indicating a potential cosmeceutical activity of this microalgal species.

Reactive oxygen species (ROS) have been linked to the pathogenesis of several human diseases such as atherosclerosis, diabetes mellitus, chronic inflammation, neurodegenerative disorders and many types of cancers. ROS species can be partially neutralized by antioxidant compounds that can reduce the risk of many diseases related to oxidative stress^1^. Hence, consumer preference for natural products is increasing the interest in finding new antioxidants from natural sources because synthetic products can cause potential long term toxic effects^2^. Most, if not all, commercially available natural antioxidants are derived from terrestrial plants (e.g. rosemary, tea, coffee, grape seeds, tomato and cocoa). Many of these antioxidants are carotenoids that are a class of more than 700 naturally occurring pigments synthesized by plants, algae, and photosynthetic bacteria. Carotenoids are known to be potent physical and chemical quenchers of singlet oxygen (^1^O_2_) and scavengers of other reactive oxygen species (ROS). However, the exact mechanisms underlying the protective function and specific molecular targets of carotenoids *in vivo* and *in vitro* are still poorly understood^3^.

*Tetraselmis suecica* is a marine green microalga belonging to the class Chlorophyceae, widely used in aquaculture for the feeding of mollusks and crustacean larvae^4^ and as a probiotic in fish^5^. *T. suecica* is rich in vitamin E, carotenoids, chlorophyll, and tocopherols^6^ and has been suggested as a food supplement in human and animal diets^7^. The total pigment extract from *T. suecica* has been patented for its ability to enhance dermal pigmentation, reduce psoriasis lesions and increase hair growth^7^. Here we investigate the potential biotechnological application of this species studying the protective role at molecular level on human anaplastic cells and tissues. To this aim, we characterize the pigment content of an ethanol/water extract of *T. suecica* and investigate the antioxidant and protective effects of this extract against oxidative damage. We show that this extract has a strong antioxidant and cell repairing activity in a human lung cancer cell line (A549), an *in-vitro* model that is often used to study antioxidant effects^8^. In particular, this total extract targets the expression of dehydrocholesterol reductase-24 (DHCR24) and prostaglandin reductase 2 (PTGR2) genes and proteins, and reduces the levels of prostaglandin E_2_ (PGE_2_). Finally, the cell repairing effect of this extract is demonstrated in *in vitro* reconstructed human epidermal tissue cells (EpiDerm^TM^) indicating a potential cosmeceutical activity of this microalgal species.

## Results

**Figure 1:**
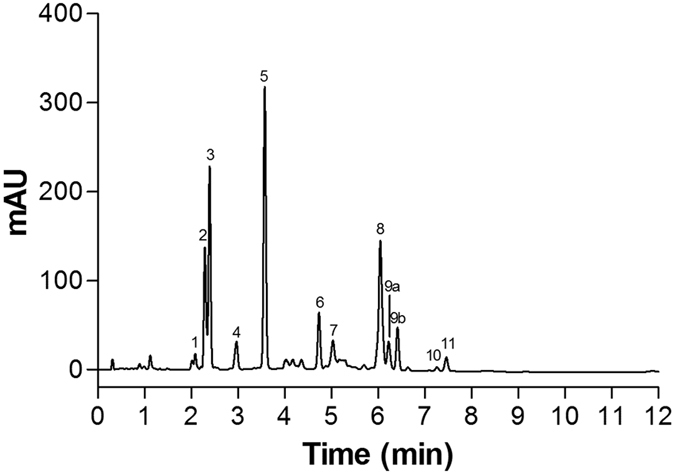
HPLC chromatogram of pigments from an ethanol/water extract of the green microalga *Tetraselmis suecica.*

The high performance liquid chromatography (HPLC) pigment profile of the ethanol/water extract of *T. suecica* revealed porphyrin pigments, chlorophyll *a* and *b*, α-and γ-carotene and xanthophyll pigments such as lutein, loroxanthin dodecenoate, violaxanthin, neoxanthin, 9′-*cis*-neoxanthin and antheraxanthin (Fig. 1, Table 1). The pigments were identified by diode array (DAD) spectroscopy and comparing their visible absorption spectra with authentic standard.

**Figure 2:**
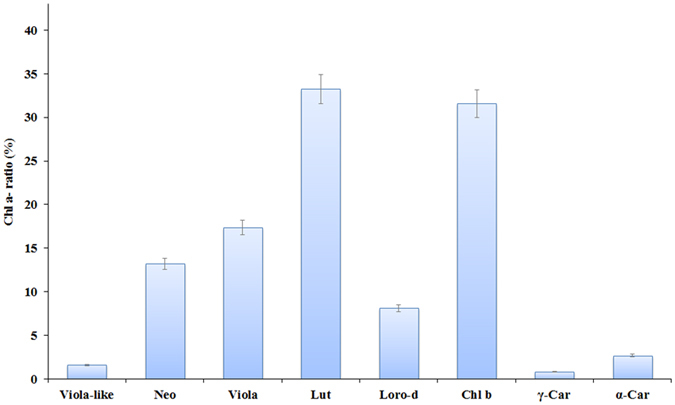
Pigment content of *Tetraselmis suecica* ethanol/water extract. Content data of 9′-*cis*-Neoxanthin and Antheraxanthin are missing due to their co-elution. Data are reported as percentage of pigment/chlorophyll a ratio.

Results were also supported by HPLC-PDA-MS/MS data (Supporting Information, Figure S1, Table S1). Other xanthophyll pigments, such as loroxanthin and zeaxanthin, which are usually found in green algae, were not observed. Loroxanthin decenoate was tentatively identified only by LC-MS/MS analysis. Xanthophylls constituted almost 79% of the total pigments identified, and, within the group, lutein was the most abundant, reaching concentrations comparable to that of chlorophyll *b* (Chl *b* 31% of Chl *a*, Lutein 33% of Chl *a*, Fig. 2). Neoxanthin and violaxanthin pigments showed a percentage over Chl *a* of about 16%, whereas loroxanthin dodecenoate a percentage of 8%. This ethanol/water extract (herewith referred to as extract) exhibited marked reducing activity toward radical species when the 2,2-diphenyl -1-picrylhydrazyl (DPPH) radical scavenging ability was tested. Addition of extract concentrations of 25, 50 and 100 μg resulted in a dose-dependent reduction (21.5%, 52.0% and 97.7%, respectively) of the purple radical DPPH into the yellow reduced form. This activity was significantly stronger than the positive control, α-Tocopherol, tested at the same concentrations (Table 2).

**Figure 3:**
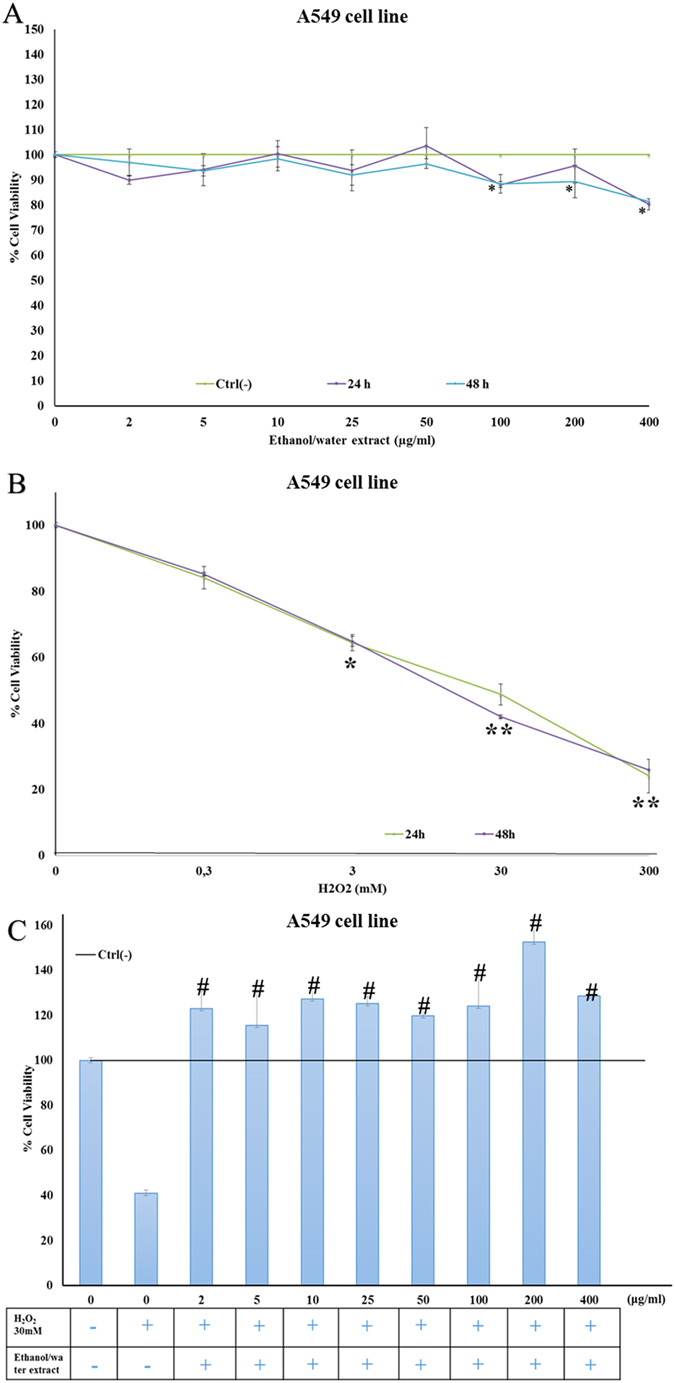
*In vitro* repairing activity of *Tetraselmis suecica* ethanol/water extract against H_2_O_2_ treatment. (**A**) Human lung adenocarcinoma cells (A549) treated with various concentrations of *T. suecica* extracts for 24 and 48 h. Cell viability was determined using the MTT assay and expressed as the percentage of control growing cells. (**B**) Cell viability of lung adenocarcinoma cells (A549) treated with various concentrations of H_2_O_2_ (0.3, 3, 30, 300 mM) for 24 and 48 h. (**C**) Effect of extract on cell viability of A549 cells following exposure to H_2_O_2_ prior to extract treatment at 2, 5, 10, 25, 50, 100, 200 and 400 μg ml^−1^. Three independent assays were performed in triplicate; data are shown as mean ± S.D. Significant differences between treated groups were determined using Students-t test (*) and ANOVA followed by Dunnett’s test (#). Cross-hatched symbols denote significant differences between treatments and control (^#^p < 0.05).

Lung adenocarcinoma (A549) cells treated with different concentrations of the extract for 24 and 48 h were not affected at any of the concentrations tested (2, 5, 10, 25, 50, 100, 200 μg ml^−1^) except for A549 cells at the highest concentration, which induced a slight reduction in cell viability (80 and 81% cell viability, at 400 μg ml^−1^, at the two incubation times, Fig. 3A).

In order to assess the antioxidant effects of the extract we induced an oxidative stress on A549 cells with hydrogen peroxide (H_2_O_2_). First, we treated A549 cells with a wide range of H_2_O_2_ concentrations (0.3, 3, 30 and 300 mM) to determine the half maximal Inhibitory Concentration (IC50) dose for pretreatment of cells, before recovery experiments with extract; the IC50 dose was established as 30 mM after 24 h and 48 h of treatment (Fig. 3B). We then treated A549 cells with 30 mM of H_2_O_2_ for 1 h, inducing H_2_O_2_ oxidative stress, and added the extract to cells. The extract induced a significant recovery effect on H_2_O_2_ stressed A549 cells after 48 h of treatment. Histograms in Fig. 3C show the effect of treatment with H_2_O_2_ (injury treatment) and extract. Treatment with H_2_O_2_ resulted in 36% cell viability after 48 h, whereas addition of extract induced a proliferation of A549 cells, with a cell viability increase of 143, 114, 125, 112, 133, 126, 114 and 148% for all concentrations tested (2, 5, 10, 25, 50, 100, 200 and 400 μg ml^−1^) with respect to the negative control. Within the experimental error, there was a plateau effect already at 2 μg ml^−1^ of the extract and a further increase of this concentration did not affect cell viability.

**Figure 4:**
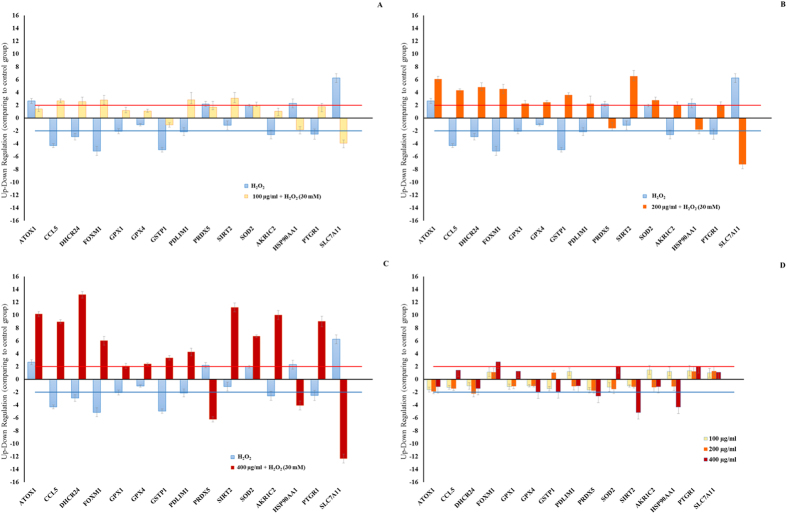
(**A**,**B**,**C**) Effect of *Tetraselmis suecica* ethanol/water extract at three different concentrations (100, 200 and 400 μg ml^−1^) on oxidative stress gene expression in H_2_O_2_-treated human lung adenocarcinoma cells (A549). A549 cells were pretreated with H_2_O_2_ (30 mM = 12 μg ml^−1^) for 1 h prior to extract treatments (100, 200 and 400 μg ml^−1^) and harvested 2 h later. **(D)** Negative control for the evaluation of the effect of *Tetraselmis suecica* ethanol/water extract at three different concentrations (100, 200 and 400 μg ml^−1^) on oxidative stress gene expression in human lung adenocarcinoma cells (A549). Three independent assays were performed in triplicate and the data are expressed as mean ± S.D. Expression values greater or lower than a two-fold difference with respect to the controls were considered significant.

The expression of genes involved in oxidative stress and repairing pathways (Supplementary information, Table S2) were analyzed in A549 cells treated with 100, 200 and 400 μg ml^−1^ of extract alone (Fig. 4D) and 100, 200 and 400 μg ml^−1^ of extract after 1 h of exposure to 30 mM of H_2_O_2_ (Fig. 4A,B and C respectively) since lower extract concentrations did not induce changes in gene and protein expression. Gene expression results are reported after 2 h of treatment since several factors implicated in oxidative damage repairing pathways were already expressed and activated after this time interval. Control genes for real-time qPCR were actin-beta (ACTB), beta-2-microglobulin (B2M), hypoxanthine phosphoribosyltransferase (HPRT1) and ribosomal protein large P_0_ (RPLP_0_), the expression of which remained constant. For gene expression studies, we chose the three highest concentrations (100, 200 and 400 μg ml^−1^) that showed strong repairing activities even if we observed a slight cytotoxicity (about 20%) at the two highest concentrations (200 and 400 μg ml^−1^). Notwithstanding the toxicity, there was a significant dose-dependent activation of specific oxidative stress response mechanisms without activating genes involved in cell death programs with all three concentrations. This suggests that the slight cytotoxicity did not inhibit the repairing activity of the extract.

24-dehydrocholesterol reductase (DHCR24) was down-regulated only at 200 μg ml^−1^ (2.2-fold change). Whereas at 400 μg ml^−1^ all the following genes were down-regulated: glutathione peroxidase 4 (GPX4, 2.0-fold change), glutathione *S*-transferase pi 1 (GSTP1, 2.0– fold change), peroxiredoxin 5 (PRDX5, 2.6-fold change), sirtuin 2 (SIRT2, 5.18-fold change) and heat shock protein alpha class A member 1 (HSP90AA1, 90 kDa, 4.3-fold change). At the same concentration, there was an up-regulation of forkhead box M1 (FOXM1, 2.71-fold change), superoxide dismutase (SOD2, 2.0-fold change) and prostaglandin reductase 1 (PTGR1, 2.0-fold change). As shown in Fig. 4A,B and C, the antioxidant protein 1 homolog gene (ATOX1) was up-regulated after H_2_O_2_ treatment (2.7-fold change) and was highly up-regulated after extract recovery treatment at 200 and 400 μg ml^−1^ (6.1 and 10.2-fold change, respectively). The small inducible cytokine subfamily A5 gene (CCL5) was down-regulated with H_2_O_2_ (4.3-fold change) and was up-regulated with the extract at all concentrations tested (2.7, 4.3 and 8.9-fold change, respectively). Opposing gene expression patterns between H_2_O_2_ and extract recovery treatment at all concentrations tested were recorded in: 24-dehydrocholesterol reductase (DHCR24, −2.9 vs 2.5, 4.8 and 13.2-fold change), forkhead box M1 (FOXM1, −5.1 vs 2.5, 4.5 and 6.0-fold change), glutathione peroxidase 1 (GPX1 −2.0 vs 1.2, 2.0 and 2.1-fold change) and glutathione peroxidase 4 (GPX4, −1.04 vs 1.0, 2.5 and 2.4-fold change, respectively), glutathione *S*-transferase pi 1 (GSTP1, −4.9 vs −1.1, 3.6 and 3.4-fold change), C-terminal lim domain protein 1 (PDLIM1, −2.11 vs 2.8, 2.2 and 4.3-fold change) and aldo-keto reductase family 1, member C2 (AKR1C2, −2.6 vs 1.1, 2.03 and 10.0-fold change). On the contrary, peroxiredoxin 5 (PRDX5), solute carrier family 7 (SLC7A11) and heat shock protein alpha class A member 1 (HSP90AA1, 90 kDa) were up-regulated with H_2_O_2_ (2.2, 6.2 and 2.3-fold change) and down-regulated or poorly expressed at 100 μg ml^−1^ (1.7, −1.6 and 6.0-fold change), 200 μg ml^−1^ (−1.6, 7.2 and −1.8-fold change) and 400 μg ml^−1^ (−6.0, −12.3 and −4.1-fold change). Interestingly, prostaglandin reductase 1 (PTGR1) was down-regulated after H_2_O_2_ injury (−2.5-fold change) and was even up-regulated after extract treatment at all concentrations (1.8, 2.0 and 9.0-fold change). Gene expression of the sirtuin 2 (SIRT2) and superoxide dismutase (SOD2) was enhanced by extract treatment after 1 h of exposure to H_2_O_2_ (from −1.1 to 3.1, 6.5 and 11.2, and from 1.9 to 2.0, 2.8 and 6.7-fold change, respectively).

**Figure 5:**
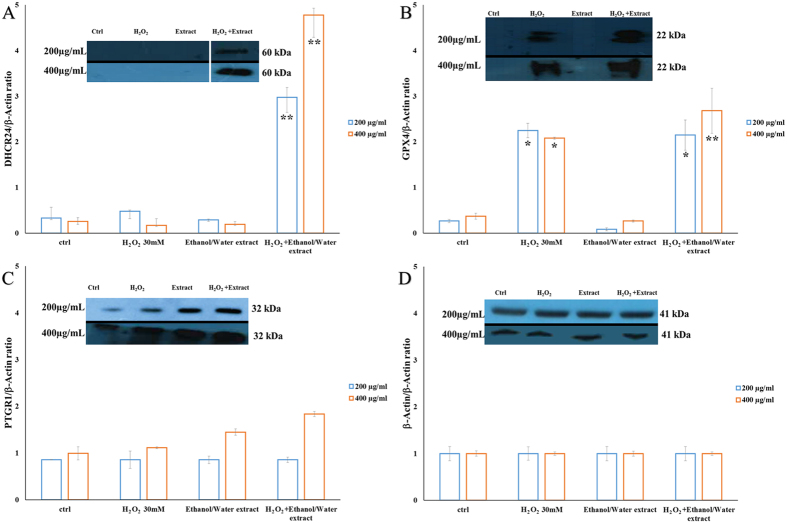
The effect of *Tetraselmis suecica* ethanol/water extract on oxidative stress protein expression in H_2_O_2_-treated human lung adenocarcinoma cells (A549). Three independent assays were performed in triplicate and the data shown are mean ± S.D. The values above the blots represent the densitometric analysis of the photographic sheets measuring the variation in protein expression. The values of the bands are normalized versus actin and represented as ratio between the expression of single protein and actin. Asterisks denote significant differences compared to controls (*p ≤ 0.05 and **p < 0.005).

**Figure 6:**
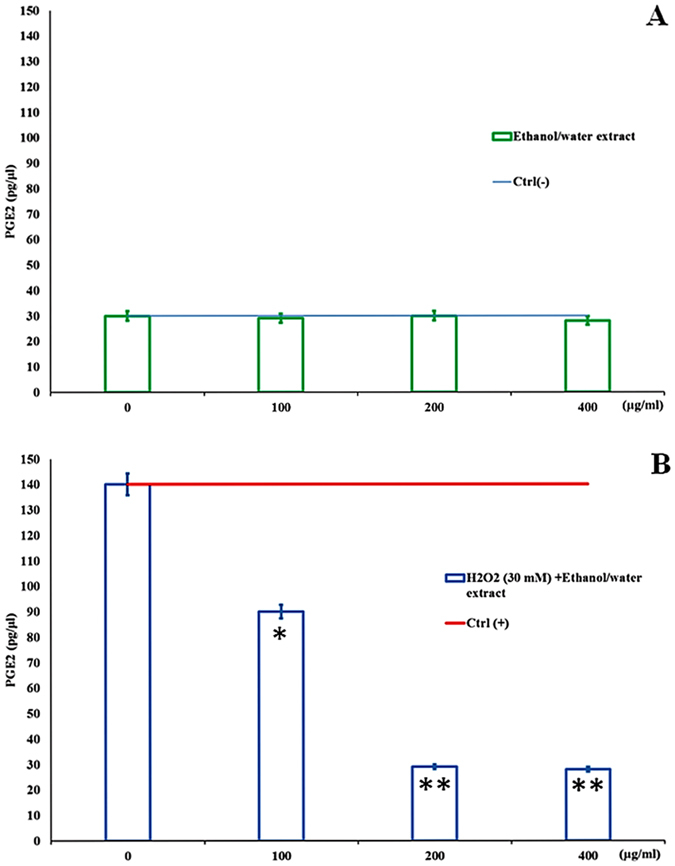
The effect of ethanol/water extract from *Tetraselmis suecica* on prostaglandin PGE_2_ serum-release induced by H_2_O_2_ -treatment in human lung adenocarcinoma cells (A549). (**A**). Average PGE_2_ concentration (pg μl^−1^) determined by ELISA in culture medium of cells treated with 100, 200 and 400 μg ml^−1^ of the extract for 24 h. (**B**). Average of the PGE_2_ concentration (pg μl^−1^) determined by ELISA in culture medium of cells treated with 100, 200 and 400 μg ml^−1^ of extract for 24 h after pretreatment with 30 mM ( = 12 μg ml^−1^) of H_2_O_2_ for 1 h. Asterisks denote significant differences compared to controls (*p ≤ 0.05 and **p < 0.005) and determined using Students-t test.

Since DHCR24, GPX4 and PTGR1 genes play crucial roles in antioxidant/anti-inflammatory cell signaling pathways, A549 cells were treated with 200 and 400 μg ml^−1^ extract for 24 h in the presence and absence of 30 mM H_2_O_2_ and protein levels were analyzed by immunoblot. We chose the two most active concentrations used for PCR array analysis in order to compare the upregulation between gene and protein expression. Exposure time was 24 h since protein expression levels were too low before this time. Immunoblot analysis revealed a significant increase only in the expression of DHCR24 at the two concentrations tested (Fig. 5A), whereas the expression of GPX4 and PTGR1 significantly increased only after H_2_O_2_ pretreatment at 400 μg ml^−1^ (Fig. 5B,C). In light of these results, we hypothesized that the extract was able to repair peroxidative cell damage by reducing the quantity of prostaglandins. Quantitative ELISA test was used to determine prostaglandin E_2_ (PGE_2_) levels secreted by A549 cells in cell culture medium before and after extract treatment. As shown in Fig. 6A, A549 cells treated with extracts (100, 200 and 400 μg ml^−1^ concentrations) had the same levels of prostaglandin E_2_ (29, 30 and 28 pg μl^−1^, respectively) as the negative control (30 pg μl^−1^). On the contrary, there was a significant dose-dependent decrease (90, 29 and 28 pg μl^−1^) in prostaglandin E_2_ levels with respect to the positive control (140 pg μl^−1^) in A549 cells treated with extract (100, 200 and 400 μg ml^−1^) after pretreatment with 30 mM of H_2_O_2_ (Fig. 6B). Our data show that extract treatment results in a significant decrease in PGE_2_ levels in cells damaged by H_2_O_2_.

**Figure 7:**
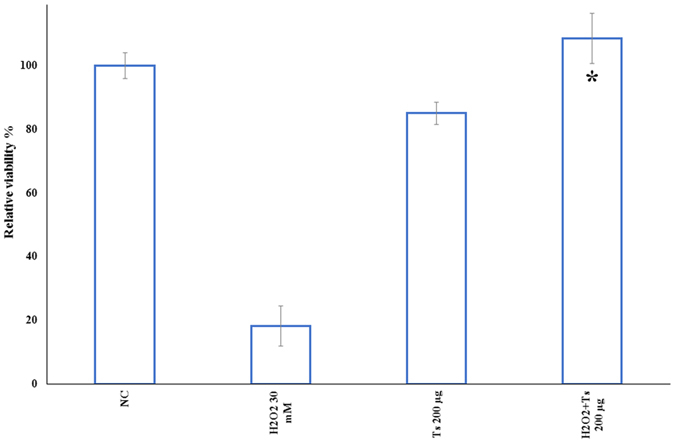
Response of EpiDerm^TM^ tissue cultures after topical application of 30 mM ( = 12 μg ml^−1^) H_2_O_2_ for 1** **h prior to *Tetraselmis suecica* ethanol/water extract treatment (200 μg ml^−1^) showing the repairing effect of the extract after H_2_O_2_ treatment. Three independent assays were performed in triplicate; data are shown as mean ± S.D. Significant differences between treated groups were determined using Students-t test (*p ≤ 0.05) and ANOVA. NC = not treated. Ts 200 μg = 200 μg *T. suecica* extract; H_2_O_2_ + Ts 200 μg = epidermal tissue pretreated with H_2_O_2_ for 1 h and recovered with extract.

Finally, we used the reconstructed human epidermal tissue model EpiDerm EPI-200 (size 0.63 cm^2^) as *in vitro* model to confirm the potential application of this extract as cosmeceutical. In particular, we chose this tissue because *in vivo* oxidative stress frequently occurs in the epidermidis causing aging and other oxidative stress-related diseases. EPI-200 was treated with 30 mM H_2_O_2_ and with 200 μg ml^−1^ of the ethanol/water extract for 1 h, after injury with H_2_O_2_. The epidermal tissue model was treated with 200 μg ml^−1^ of the extract since this was the lowest concentration at which gene and protein expression data revealed a complete activation of all key factors in the antioxidant pathway. Treated medium was removed and replaced with extract, but without H_2_O_2_, to assess if the irritant effect persisted after 24 h of recovery (referred to as recovery time). Treatment with 200 μg ml^−1^ extract for 1 h significantly affected tissue viability after 24 h recovery time (85% viability) compared to the irritant effect of 1 h treatment with H_2_O_2_ (18% viability) (Fig. 7). The repairing effect by the extract was even more evident 1 h later after treatment with 30 mM H_2_O_2_ and 200 μg ml^−1^ of the extract tissue, with viability increasing from about 20% to 108.5% with respect to the negative control.

## Discussion

Previous studies have shown that total extracts of *Tetraselmis* sp. have potential cosmetic and pharmaceutical applications for the human hair growth and pigmentation of the skin, and also for stimulating the increased production of skin structural proteins such as filaggrin and involucrin involved in dermal diseases as Psoriasis^8^. In a previous study, an ethanol/water extract of *Tetraselmis suecica* showed a strong scavenging activity against 2,2-difenyl-1-picrylhydrazyl (DPPH) and peroxyl radicals rather than against the superoxide anions from the xanthine/xanthine oxidase system^9^. Here we show that a similar type of extract from *T. suecica* also stimulates a strong response to cell damage and activates a repairing mechanism in human epidermal cells. Our extract contains high levels of xanthophylls (lutein, violaxanthin, neoxanthin, antheraxanthin and loroxanthin esters), pigments that are well known for their biological activities as antioxidants and which are precursors of other pigments or vitamins^10^. Lutein, which was particularly abundant in this extract, possesses pronounced free radical scavenging ability due to its polarity and number of conjugated double bonds^11^, and has been shown to significantly decrease neurogenic inflammatory response in the mouse skin^12^. The antioxidant activity of violaxanthin and neoxanthin is also well documented^13^, whereas the biological activity of antheraxanthin and loroxanthin esters recently characterized in *T. suecica*^14^ have not yet been investigated.

In our study, the ethanol/water extract, containing high levels of carotenoids, from *T. suecica* showed marked radical scavenging ability when tested with the DPPH assay. The addition of extract led to 98% reduction of the radical DPPH (purple) into its reduced (yellow) form at the highest concentrations. As reported in Table 2, the radical scavenging activity of the extract was dose-dependent and its strength was 70% greater than α-Tocopherol at the highest concentrations. Moreover, the inhibition of DPPH free radicals induced by the extract was comparable to other well-known antioxidant molecules such as ascorbic acid where the percentage reduction of the free radical is about 95%^15^.

In order to evaluate this effect at the cellular level we challenged cells with H_2_O_2_ because a previous study showed that A549 lung cells have a multifaceted response when exposed to hydrogen peroxide^16^. D’Andrea *et al*.^16^ have shown that H_2_O_2_ induces damage to lipids, proteins, and nucleic acids due to the generation of reactive oxygen species (ROS). In our case, H_2_O_2_ caused a reduction in A549 cell viability to 36% after 48 h, but addition of the extract induced a strong recovery in cell viability, with up to 100% recovery in some cases. In order to clarify this effect at the molecular level, we studied the difference in oxidative stress gene expression patterns between cells treated only with 30 mM H_2_O_2_ and cells recovered with 100, 200 and 400 μg ml^−1^ extract. The genes involved in ROS metabolism such as oxidative stress responsive genes ATOX1, CCL5 (RANTES), DHCR24, FOXM1, GPX1, GPX4, PDLIM1, PRDX5, SIRT2, SOD2 were all significantly up-regulated in a dose dependent manner after extract recovery treatment for the highest concentrations tested.

In particular, antioxidant genes such as GPX1 and GPX4 showed a completely reversed expression after extract-induced recovery with respect to H_2_O_2_ injury for all concentrations tested. On the contrary, PRDX5 showed an up-regulation with H_2_O_2_, because this gene codes for a mitochondrial peroxiredoxin^17^ that decomposes hydrogen peroxide. However, after recovery treatment with extract, PRDX5 gene expression levels decreased in a dose dependent manner and were significantly down-regulated at 400 μg ml^−1^. This interesting finding is probably due to the ability of the extract to scavenge the effect induced by H_2_O_2_ thereby reducing PRDX5 gene expression levels.

The genes involved in ROS metabolism such as SOD2 were up-regulated with H_2_O_2_. Recovery treatment with the extract caused an enhanced up-regulation of this gene in a dose dependent manner. This is an important finding because, as demonstrated in previous studies, mitochondrial SOD2 plays a crucial role in protecting cells against oxidative stress^18^. Another important result regards the effect of the extract on up-regulation of DHCR24 because this is a multifunctional enzyme, which exerts resistance against oxidative stress and prevents apoptotic cell death when it is expressed at high levels^19^. The increase in expression levels of the inflammatory pathway gene PTGR1 suggests a potential anti-inflammatory activity because this enzyme is responsible for the biological inactivation of prostaglandins and related eicosanoids^20^.

The down-regulation of the GSTP1 gene after H_2_O_2_ treatment indicates that cells were unable to defend themselves against injury. Surprisingly, after 200 and 400 μg ml^−1^ of the recovery treatment with the extract, GSTP1 was significantly up-regulated indicating the restoration of the antioxidant defense mechanisms.

Peroxide metabolism genes such as AKR1C2, HSP90AA1 and SLC7A11 showed different expression patterns. In particular, the AKR1C2 gene, which catalyzes the conversion of aldehydes and ketones to their corresponding alcohols^21^, was down-regulated with H_2_O_2_ treatment and up-regulated in a dose-dependent manner by the recovery treatment with the extract (only for 200 and 400 μg ml^−1^). The activation of this gene indicates a specific cell response to hydrogen peroxide metabolites through the induction of detoxification mechanisms. Heat shock protein 90 kDa alpha (cytosolic) class A member 1 (HSP90AA1) was up-regulated by H_2_O_2_ treatment because it is a pro-apoptotic factor which induces cell death in response to stress. The extract was able to down-regulate HSP90 expression leading to the induction of cytoprotective pathways through the inhibition of pro-apoptotic pathways^22^. Surprisingly, the extract was able to down-regulated the SLC7A11 gene which is up-regulated by the H_2_O_2_ treatment. SLC7A11 encodes a subunit of the xCT cystine/glutamate aminoacid transport system, which is involved in the generation of glutathione and the protection of cells from oxidative stress. However, in a recent study the expression of SLC7A11 was shown to promote tumorigenesis and chemotherapy resistance^23^. The down-regulation of SLC7A11 by the extract may therefore be considered as a potential chemo-preventive agent.

In order to demonstrate the activation of an oxidative stress response pathway after *T. suecica* extract recovery treatment, we also analyzed the expression of key proteins involved in antioxidant mechanisms (GPX4 and DHCR24) and PTGR1. Immunoblot data confirmed gene expression results on the induction of an antioxidant pathway in A549 cells damaged with H_2_O_2_ and then treated with extract (Schematic representation of the oxidative stress response pathway in Supplementary Information, Figure S3). We found that the physiological increase of the active form of GPX4 protein after 30 mM H_2_O_2_ treatment was enhanced by recovery treatment with 200 and 400 μg ml^−1^ extract. Another important finding was the increased expression of DHCR24 and PTGR1 proteins induced by extract treatment. The high up-regulation of PTGR1 could be linked to a reduction in prostaglandin release by cells. ELISA experiments showed a significant dose-dependent decrease in prostaglandin PGE_2_ levels in the culture medium after recovery treatment confirming this hypothesis. To our knowledge, this is the first report that an ethanol/water extract from a marine green microalga acts as an inhibitor of prostaglandin release in an inflammatory response.

Due to difficulties in obtaining sufficient human lung epithelial cells to perform this study, we chose the A549 cell line derived from a lung carcinoma due to its considerable use in the literature as a surrogate cell type^24^,^25^,^26^,^27^ due to its high levels of glutathione^28^ and high (non-induced) heme oxygenase 1 (HO-1) gene expression levels^29^. We are aware that results obtained from this transformed cell line may not be applicable and immediately transferrable to normal lung epithelial cells in the respiratory tract *in vivo*. Thus, in order to use this extract as a potential cosmetic agent for topical application, we used human epidermidis tissue (EPI-200) as an experimental model and showed that the extract exerted a strong repairing effect after injury caused by hydrogen peroxide.

To date, intervention trials with single antioxidants in pharmacological doses have not supported a repairing effect in humans^12^. However, if many antioxidants work in a network, ‘total antioxidants’ may be a better concept than individual antioxidants. Thus, the potential synergistic effects of bioactive components, such as carotenoids, with different chemical structures and anti-oxidizing activities may be a promising agent for cosmeceutical use. The identification of potent marine microalgal species as peroxide scavengers and repairing agents can lead to new alternative cosmeceutical products or nutritional supplements for prevention of disorders related to oxidative damage, such as cancer, aging and skin inflammation diseases. In order to develop new natural cosmeceutical products for human health applications from marine microalgae, further studies are required to clarify if this bioactivity is ascribable to a single compound, classes of molecules (e. g. carotenoids) or the synergistic effect of several molecules contained in the ethanol/water extract of *Tetraselmis suecica*.

## Methods

### Strain and culture conditions

The prasinophyte *Tetraselmis suecica* was purchased from the American marine phytoplankton collection (CCMP 906) and was grown in Guillard’s f/2^30^ medium without silicic acid in two-liter polycarbonate bottles, constantly bubbled with air filtered through 0.2 μm membrane filters. Cultures were grown at 19 °C, a photon-flux density of about 150 μmol photons m^−2^ s^−1^, and a photoperiod of 12:12 h light:dark (12 L:12D) cycle. Initial cell concentrations were about 5 × 10^3^ cells ml^−1^. Microalgal biomass was collected by centrifugation after 9 days (~8 × 10^3^ cells ml^−1^).

### Preparation of ethanol/water extract from *Tetraselmis suecica*

Extraction was performed according to Goiris *et al*.^31^. Extraction procedure was conducted under dark conditions, at room temperature and under nitrogen atmosphere, in order to avoid oxidation of the sample. Freeze-dried biomass (~100 mg) was extracted with 1 ml ethanol/water (3/1 v/v) mixture for 30 min. The mixture was separated by centrifugation at 4500 × *g*, for 10 min, at 20 °C, and the upper layer was transferred to a clean tube. The pellet was resuspended in 1 ml of the ethanol/water mixture and extracted for a second time. The ethanolic extract was dried in a rotary vacuum evaporator (Buchi rotavapor R-114). Dry extract was stored under nitrogen atmosphere at −20 °C prior to analysis.

### HPLC analysis of bioactive pigments

Pigment measurements were conducted by High Performance Liquid Chromatography (HPLC) on an aliquot of the ethanolic extract (10 mg), according to methods described in^32^. Prior to injection into the HPLC, 250 μL of an Ion Pairing Agent (ammonium acetate 1 mol L^−1^, final concentration of 0.33 mol L^−1^) was added to 0.5 mL of the pigment extract and incubated for 5 minutes in the dark at 4 °C. This extract was then injected in the 50 μL loop of the Hewlett Packard series 1100 HPLC (Hewlett Packard, Wilmington, NC, USA), equipped with a reversed-phase column (C8 Kinetex column; 50 mm × 4.6 mm; 2.6 μm particle size, Phenomenex^®^, USA). The temperature of the column was steadily maintained at 20 °C, and the flow rate of the mobile phase was set up at 1.7 mL min^−1^. The mobile phase was composed of two solvent mixtures: A, methanol/aqueous ammonium acetate (70/30, v/v) and B, methanol. During the 12-minutes elution, the gradient between the solvents was programmed: 75% A (0 min), 50% A (1 min), 0% A (8 min) isocratic for 3 min. Chlorophylls and carotenoids were detected by diode-array spectroscopy (spectrum data collected in the range 350–750 nm) using a Hewlett Packard photodiode array detector, model DAD series 1100 and absorbance chromatogram was reported at 440 nm. Chlorophylls were also detected by fluorescence using a Hewlett Packard standard FLD cell series 1100 with excitation and emission wavelengths set at 407 nm and 665 nm, respectively. Identification and quantification of pigments were carried out using pigment standards from the D.H.I. Water & Environment (Horsholm, Denmark). Pigment standards derived primarily from phytoplankton. The standards are flushed with 100% N2 and supplied in sealed vials with 2.5 mL, together with a certificate of analysis. All information regarding the accuracy of the preparation of the standards and reference numbers of all standards are available at the following address: http://c14.dhigroup.com/productdescriptions/phytoplanktonpigmentstandards. Moreover spectral information was compared with a library of chlorophyll and carotenoid spectra of pigments prepared from standard phytoplankton cultures^33^.

### LC-MS/MS analysis of bioactive pigments

LC-MS/MS analysis was carried out on a Waters Alliance HPLC with a Waters 996 PDA detector on line with a Q-Tof mass spectrometer (Waters) featured by an ESI source in positive ionization mode. Column: Phenomenex Luna C8 250 × 4.6 mm, 5 μm, 100 A. Eluent A: Water, B: MeOH. Gradient: 90% B to 100% B in 15 mins, holding for 20 mins. Flow: 0.7 ml min^−1^. PDA: 400–700 nm. Full Mass range: 450–1000 *m/z*.

### Scavenging activity against DPPH radical

2,2-Di(4-tert-octylphenyl)-1-picrylhydrazyl (DPPH) was used for the radical scavenger assay (Sigma Aldrich, cat. 257621). Various concentrations of extract from *T. suecica* were mixed with a final concentration of DPPH of 0.1 mM in methanol, allowed to react for 30 min in the dark, and absorbance was measured at 517 nm using a microplate reader.

### Treatment of Human Cells

The adenocarcinomic human alveolar basal epithelial cell line A549 was purchased from the American Type Culture Collection (ATCC^®^ CCL185™) and grown in DMEM-F12 (Dulbecco’s modified Eagle’s medium) supplemented with 10% fetal bovine serum (FBS), 100 units ml^−1^ penicillin and 100 μg ml^−1^ streptomycin in a 5% CO_2_ atmosphere at 37 °C. A549 cells (2 × 10^3^ cells well^−1^) were seeded in a 96-well plate and kept overnight for attachment. The extract was dissolved in dimethyl sulfoxide (DMSO) and used for the treatment of cells. Seventy percent confluent cells were treated with extract at 2, 5, 10, 25, 50, 100, 200 and 400 μg ml^−1^ for 24 and 48 h in complete cell medium. The final concentration of DMSO used was 1% (v/v) for each treatment.

### Cell Viability

The effect of ethanol/water extract on cell viability was determined using the 3-(4,5-Dimethylthiazol-2-yl)-2,5-Diphenyltetrazolium Bromide (MTT) assay (Applichem A2231) in according to^34^. A549 cells, seeded in 96-well plates (2 × 10^3^ cells/well), after treatment times, were treated with 10 μl (5 mg ml^−1^) of MTT and incubated for 3 h. The absorbance was recorded on a microplate reader at a wavelength of 570 nm (Multiskan FC, THERMO SCIENTIFIC). The effect of the extract on cell viability was evaluated as percent of cell viability calculated as the ratio between mean absorbance of each sample and mean absorbance of control.

### RNA Extraction and Real-Time PCR

A549 cells (2 × 10^6^), used for RNA extraction and analysis, were seeded in Petri dishes (100 mm diameter) to obtain four types of samples: negative control without any treatment, positive control with 30 mM of H_2_O_2_, cells treated with ethanol/water extracts (100, 200 and 400 μg ml^−1^), and cells recovered with extracts (100, 200 and 400 μg ml^−1^) after pre-treatment for 1 h with 30 mM H_2_O_2_. After 2 h of exposure time, A549 cells were washed directly in the Petri dish by adding cold Phosphate-Buffered Saline (PBS) and rocking gently.

Cells were lysed in the Petri dish by adding 1 ml of Trisure Reagent (Bioline, cat. BIO-38033) per 100 mm dish diameter. RNA was isolated according to the manufacturer’s protocol. RNA concentration and purity was assessed using the nanophotomer NanodroP (Euroclone).

About 200 ng RNA was subjected to reverse transcription reaction using the RT^2^ first strand kit (Qiagen, cat.330401) according to the manufacturer’s instructions. The qRT-PCR analysis was performed in triplicate using the RT^2^ Profiler PCR Array kit (Qiagen, cat.330231), in order to analyze the expression of cell oxidative stress genes on A549 cells. Plates were run on a ViiA7 (Applied Biosystems 384 well blocks), Standard Fast PCR Cycling protocol with 10 μl reaction volumes. Cycling conditions used were: 1 cycle initiation at 95.0 °C for 10 min followed by amplification for 40 cycles at 95.0 °C for 15 s and 60.0 °C for 1 min. Amplification data were collected via ViiA 7 RUO Software (Applied Biosystems). The cycle threshold (Ct)-values were analyzed with PCR array data analysis online software (http://pcrdataanalysis.sabiosciences.com/pcr/arrayanalysis.php, Qiagen).

### Protein Extraction and Western Blotting

A549 cells (2 × 10^6^), used for protein extraction and analysis, were seeded in Petri dishes (100 mm diameter) to obtain four types of samples: negative control without any treatment, positive control with cells treated with 30 mM H_2_O_2_, cells treated with 100, 200 and 400 μg ml^−1^, and cells recovered with 100, 200 and 400 μg m^−1^ of extract after pre-treatment for 1 h with 30 mM H_2_O_2_. A549 cell lysate was prepared after 24 h of treatment by scraping the cells of each Petri dish into 1 ml of Radio Immune Precipitation Assay buffer (RIPA, Cell Signaling, cat. 9806), supplemented with 1 μM of protease inhibitor PMSF (Cell Signaling, cat. 8553). The lysate was incubated on ice for 15 min and then clarified by centrifugation at 14000 × *g*, for 20 min. Total protein concentration was determined according to the Bradford method using a Protein Assay Reagent (Applichem, cat. A6932) with bovine serum albumin (BSA, Sigma Aldrich, cat. A2058) as a standard. The protein extract was stored at −20 °C until use. Before electrophoresis, protein samples were incubated at 100 °C for 5 min. Following 10% SDS-PAGE, gels were stained with Coomassie or blotted onto nitrocellulose membrane (Biorad, cat. 170–4159). Membranes were incubated for 1 h in blocking reagent (1X Tris Buffered Saline-TBS), with 0.1% Tween-20 with 5% w/v nonfat dry milk, and incubated overnight at 4 °C with the primary antibodies diluted in 1X TBS, 0.1% Tween-20 with 5% BSA.

Three key proteins were investigated: 24-dehydrocholesterol reductase (DHCR24, 1:1000, Sigma Aldrich SAB1405713), glutathione peroxidase 4 (GPX4, 1:1000, Sigma Aldrich SAB2500486), prostaglandin reductase 1 (PTGR1, 1:1000, Sigma Aldrich SAB4500918). Positive control was obtained by using anti-β-actin antibody (1:500, Novus Biological cat. NB600-501).

After incubation, membranes were washed three times for 5 min each with 15 ml of TBS/Tween and then incubated with HRP-conjugated secondary antibody with gentle agitation for 1 h at room temperature. For β-actin and DHCR24 antibodies, we used HRP-conjugated secondary antibody anti-mouse (1:10000, Santa Cruz Biotechnology); for GPX4 and PTGR1 antibodies, we used HRP-conjugated secondary antibody anti-rabbit (1:10000, Jackson ImmunoResearch).

After incubation, membranes were washed three times for 5 min each with 15 ml of TBS/Tween. Blotted membranes were immunodetected using clarity Western ECL (Biorad, cat. 170–5060). Proteins were visualized with Fuji medical X-ray film (cat. 47410). Densitometric analysis of immunopositive bands was performed using Image J software.

### ELISA for PGE2

Prostaglandins were quantified in the cell medium by ELISA kit (Life Technologies, cat. EHPGE2) according to standard manufacturer’s recommendations. We quantified prostaglandins in four samples: A549 cells without treatment (control), A549 cells treated with 100, 200 and 400 μg ml^−1^ of extract, A549 cells treated with only 30 mM of H_2_O_2_ and A549 cells treated with 100, 200 and 400 μg ml^−1^ of the extract after pre-treatment for 1 h with 30 mM H_2_O_2_.

### Treatment of human epidermis

EpiDerm^TM^ was purchased from the MatTek Corporation (EPI-200-SIT) and maintained in culture medium provided by the manufacturer and according to a standardized protocol. The EpiDerm skin model (area of 0.6 cm^2^) consisted of normal human derived epidermal keratinocytes (NHEK) cultured to form a multilayered, highly differentiated model of the human epidermis.

EpiDerm tissues were conditioned by incubation with culture medium in order to release transport-stress related compounds and debris overnight (in a 5% CO_2_ atmosphere at 37 °C). About 200 μg ml^−1^ carotenoid extract was dissolved in dimethyl sulfoxide (DMSO, final concentration 1% v/v) and used for the treatment of human epidermis; after 1 h of incubation with the extract, a complete recovery medium was used for 24 h of recovery time.

### Tissue Viability

The effect of the extract on tissue viability was determined using the MTT assay. After 1 h treatment and 24 h of recovery time, tissues were transferred to 24-well plates containing MTT medium (1 mg ml^−1^). After 3 h incubation in MTT, the blue formazan salt was extracted with 2 ml/tissue of isopropanol and the optical density of the extracted formazan was determined using a spectrophotometer at a wavelength of 570 nm. Relative tissue viability was calculated for each tissue as percentage of the mean of the negative control tissues. Skin irritation potential of the test extract was predicted if the remaining relative tissue viability was below 50% (MatTek Corporation-Protocol).

### Statistical Analysis

Statistical significance of the DPPH assay was determined by Students-t test (*p values ≤ 0.05). Statistical differences between treated and control cells for cell viability counts were determined by One-way ANOVA and significant differences between the treated groups by Students-t test (*) and ANOVA followed by Dunnett’s test (#) (p values ≤ 0.001) using Microsoft Excel software (365 version, 2013). Gene expression data were analyzed by PCR array data analysis online software (http://pcrdataanalysis.sabiosciences.com/pcr/arrayanalysis.php, Qiagen^®^). Only expression values greater than a 2.0-fold difference with respect to the controls were considered significant. Immunoblotting protein expression was calculated as the percentage of integral area of every single gel band with respect to total gel lane area, represented as pixels. Statistical differences between treated and controls were determined by Students-t test with significant p values ≤ 0.05. Data significantly different from controls, with p values < 0.001 are marked with two asterisks in the figures. Significant differences between treated groups, after epidermal human tissue experiments, were determined using Students-t test (*p ≤ 0.05) and ANOVA.

## Additional Information

**How to cite this article**: Sansone, C. *et al*. The green microalga *Tetraselmis suecica* reduces oxidative stress and induces repairing mechanisms in human cells. *Sci. Rep.* **7**, 41215; doi: 10.1038/srep41215 (2017).

**Publisher's note:** Springer Nature remains neutral with regard to jurisdictional claims in published maps and institutional affiliations.

## Supplementary Material

Supplementary Information

## Figures and Tables

**Table 1:** Peak identification, retention time, abbreviations and online spectral characteristics.

Peak	Retention time (min)	Pigment	Abbreviation	Absorbance maxima (nm)
1	2.08	Violaxanthin-like	Viola-like	415–440–469
2	2.29	Neoxanthin	Neo	413–436–465
3	2.39	Violaxanthin	Viola	415–440–469
4	2.97	9′-*cis*-Neoxanthin + Antheraxanthin	*c*-Neo Anth	413–436–465 422–447–472
5	3.58	Lutein	Lut	418–444–474
6	4.74	Loroxanthin dodecenoate	Loro-*d*	420–448–473
7	5.04	Chlorophyll *b*	Chl *b*	457–650
8	6.05	Chlorophyll *a*	Chl *a*	430–618–665
9a	6.23	Chlorophyll *a*′	Chl *a*′	430–618–665
9b	6.42			
10	7.26	γ-Carotene	γ-Car	436–462–490
11	7.46	α-Carotene	α-Car	425–449–476

**Table 2:** Radical scavenging capacity (RSC, %) of *Tetraselmis suecica* ethanol/water extract on DPPH free radical.

Extract concentration	(μg/ml)	Optical density (OD517 nm)	Inhibition percentage (IP) of DPPH radical
ODT0-ODTS			
Tetraselmis suecica	0	0.000 ± 0.001	0.0 ± 0.789
	50	0.0655 ± 0.029	21.1 ± 2.87*
	100	0.081125 ± 0.034	52.00 ± 3.44*
	200	0.111 ± 0.051	97.72 ± 5.09*
	0	0.000 ± 0.001	0.0 ± 0.789
α-Tocopherol	50	0.0125 ± 0.052	5.98 ± 5.21
	100	0.026 ± 0.035	12.36 ± 1.10*
	200	0.040 ± 0.013	26.00 ± 1.3*

Values are reported as percentage versus a blank and are the mean ± SD of three independent samples analyzed three times. Asterisks denote significant increases in measured radical scavenging activity *p ≤ 0.05 versus control.
