# Supplementary material for: The green microalga Tetraselmis suecica reduces oxidative stress and induces repairing mechanisms in human cells
Source: Sci Rep. 2017 Jan 24;7:41215. doi: 10.1038/srep41215 (PMC5259714; doi:10.1038/srep41215)

Supplementary Information

The green microalga Tetraselmis suecica reduces oxidative stress and induces repairing mechanisms in human cells

Clementina Sansone[1], Christian Galasso[1-2], Ida Orefice[1], Genoveffa Nuzzo[3], Elvira Luongo[3], Adele Cutignano[3], Giovanna Romano[1], Christophe Brunet[1], Angelo Fontana[3], Francesco Esposito[1] and Adrianna Ianora[1].

[1]Integrative Marine Ecology Department, Stazione Zoologica Anton Dohrn, Villa Comunale, Naples 80121, Italy

[2]University of Naples "Federico II", Department of Veterinary Medicine and Animal Production, Via Federico Delpino 1, Naples 80137, Italy

[3]Bio-Organic Chemistry Unit, Institute of Biomolecular Chemistry-CNR, Via Campi Flegrei 34, Pozzuoli, Naples 80078, Italy

Figure S1-TableS1 LC-PDA-ESI$^+$MS/MS analysis of the carotenoid pool in the ethanol/water extract of *T. suecica*.

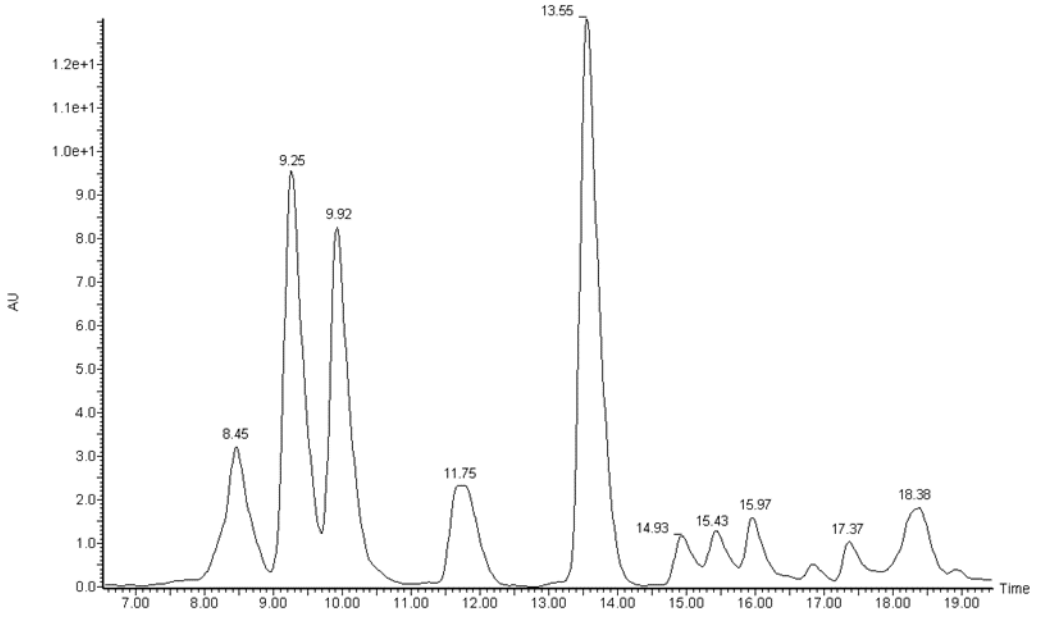

**Tr**	**Abs max (nm)**	**ESI$^+$-MS *m/z***	**ESI$^+$-MS/MS *m/z***	**Name**
**8.45**	439, 469	623.4 (M+Na^+)	605 (M-$H_2$0+Na^+), 587(M-2$H_2$0+Na^+), 531 (M-92+ Na^+)	Violaxanthin-like
**9.25**	413, 436, 464	623.4 (M+Na^+)	605 (M-$H_2$0+Na^+), 587(M-2$H_2$0+Na^+), 531 (M-92 +Na^+)	Neoxanthin
**9.92**	416, 439, 469	623.4 (M+Na^+)	605 (M-$H_2$0+Na^+), 587(M-2$H_2$0+Na^+), 531 (M-92 +Na^+)	Violaxanthin
**11.75**	440, 474	607.4 (M+Na^+)<br>+<br>623.4 (M+Na^+)	589 (M-$H_2$0+Na^+), 515 (M-92+Na^+);<br>605 (M-$H_2$0+Na^+), 587(M-2$H_2$0+Na^+), 531 (M-92+ Na^+)	Antheraxanthin<br>+<br>*c*-Neoxanthin
**13.55**	444, 472	591.4 (M+Na^+)	573 (M-$H_2$0+Na^+), 499 (M-92+ Na^+)	Lutein
**14.93**	438, 465			Not identified
**15.43**	440, 466			Not identified
**15.97**	446, 473	759.5 (M+Na^+)	589 (M-170 +Na^+)	Loroxanthin decenoate
**17.37**	446, 473	787.5 (M+Na^+)	589 (M-198 +Na^+)	Loroxanthin dodecenoate
**18.38**	457, 633	975.5 (M+Na^+)	915, 697, 665	Chlorophyll-like

Table S2. List of genes involved in the oxidative stress

Unigene	Refseq	Symbol	Description	Gname
**Oxidative Stress Responsive Genes**				
**Hs.125213**	NM_004045	ATOX1	ATX1 antioxidant protein 1 homolog	ATX1/HAH1
**Hs.514821**	NM_002985	CCL5	Chemokine (C-C motif) ligand 5	D17S136E/RANTES/SCYA5/SIS-delta/SISd/TCP228/eoCP
**Hs.498727**	NM_014762	DHCR24	24-dehydrocholesterol reductase	DCE/Nbla03646/SELADIN1/seladin-1
**Hs.239**	NM_021953	FOXM1	Forkhead box M1	FKHL16/FOXM1B/HFH-11/HFH11/HNF-3/INS-1/MPHOSPH2/MPP-2/MPP2/PIG29/TRIDENT
**Hs.76686**	NM_000581	GPX1	Glutathione peroxidase 1	GPXD/GSHPX1
**Hs.433951**	NM_002085	GPX4	Glutathione peroxidase 4	GPx-4/GSHPx-4/MCSP/PHGPx/snGPx/snPHGPx
**Hs.523836**	NM_000852	GSTP1	Glutathione S-transferase pi 1	DFN7/FAEES3/GST3/GSTP/HEL-S-22/PI
**Hs.368525**	NM_020992	PDLIM1	PDZ and LIM domain 1	CLIM1/CLP-36/CLP36/HEL-S-112/hCLIM1
**Hs.502823**	NM_181652	PRDX5	Peroxiredoxin 5	ACR1/AOEB166/B166/HEL-S-55/PLP/PMP20/PRDX6/PRXV/SBBI10/prx-V
**Hs.466693**	NM_012237	SIRT2	Sirtuin 2	SIR2/SIR2L/SIR2L2
**Hs.487046**	NM_000636	SOD2	Superoxide dismutase 2, mitochondrial	IPOB/MNSOD/MVCD6
**Peroxide metabolism genes**				
**Hs.460260**	NM_001354	AKR1C2	Aldo-keto reductase family 1, member C2	AKR1C-pseudo/BABP/DD/DD-2/DD/BABP/
**Hs.525600**	NM_001017963	HSP90AA1	Heat shock protein 90kDa alpha A	EL52/HSP86/HSP89A/HSP90A/HSP90N/HSPC1/HSPCA/HSPCAL1
**Hs.390594**	NM_014331	SLC7A11	Solute carrier family 7 member 11	CCBR1/xCT
**Antinflammatory pathway**				
**Hs.584864**	NM_012212	PTGR1	Prostaglandin reductase 1	LTB4DH/PGR1/ZADH3

Figure S3. Schematic representation of the cellular response activated by H_2O_2 (**A**) and repairing effect induced by *Tetraselmis suecica* extract (**B**) after H_2O_2 pretreatment.

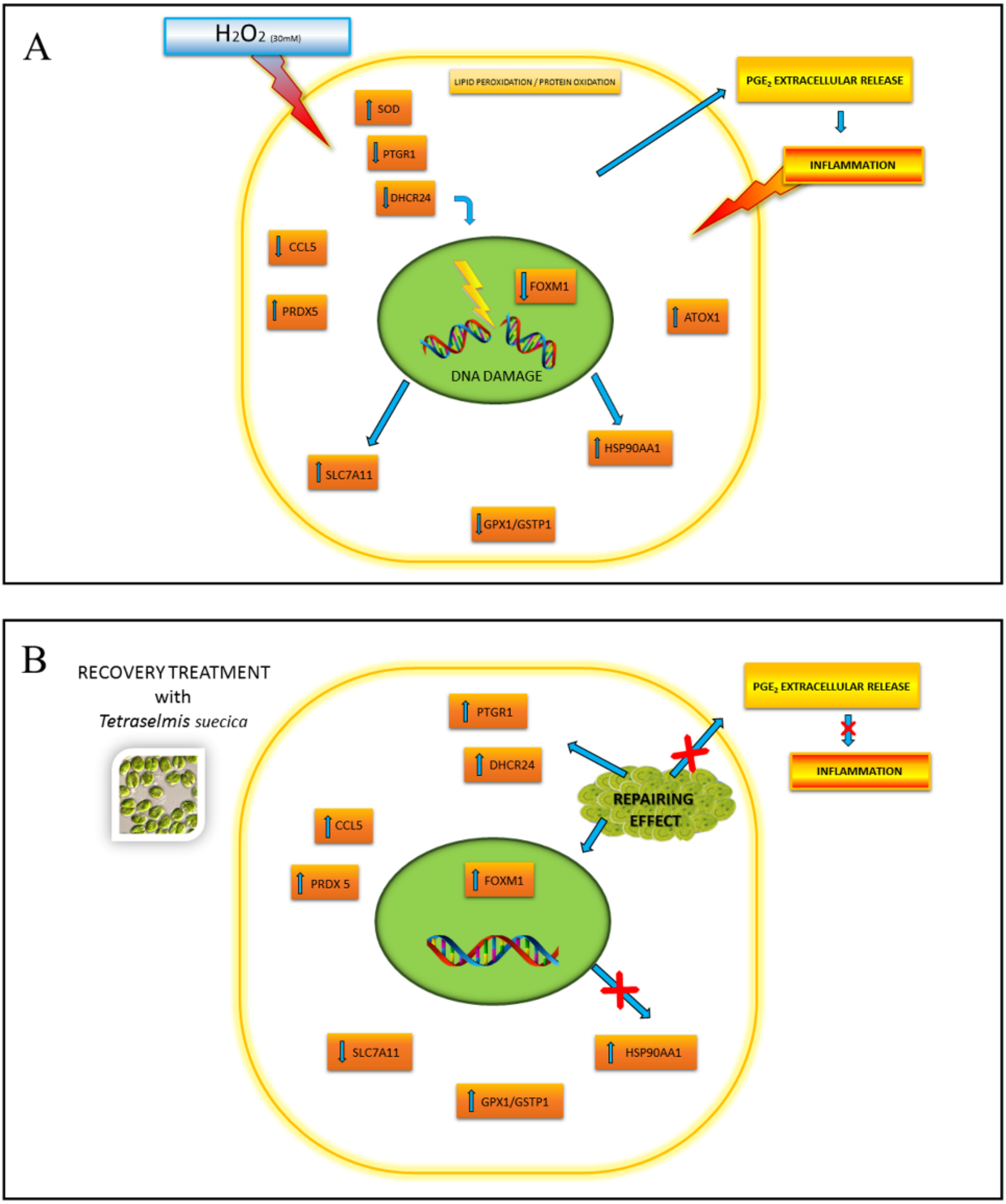

Supplement: Supplementary Information [file srep41215-s1.pdf]
